# Mesenchymal Stem Cell Secretome Enhancement by Nicotinamide and Vasoactive Intestinal Peptide: A New Therapeutic Approach for Retinal Degenerative Diseases

**DOI:** 10.1155/2020/9463548

**Published:** 2020-06-27

**Authors:** Maria L. Alonso-Alonso, Girish K. Srivastava, Ricardo Usategui-Martín, Maria T. García-Gutierrez, José Carlos Pastor, Ivan Fernandez-Bueno

**Affiliations:** ^1^Instituto Universitario de Oftalmobiología Aplicada (IOBA), Retina Group, Universidad de Valladolid, Valladolid 47011, Spain; ^2^Red Temática de Investigación Cooperativa en Salud (RETICS), Oftared, Instituto de Salud Carlos III, Valladolid 47011, Spain; ^3^Centro en Red de Medicina Regenerativa y Terapia Celular de Castilla y León, Valladolid 47011, Spain; ^4^Departamento de Oftalmología, Hospital Clínico Universitario de Valladolid, Valladolid 47003, Spain

## Abstract

Mesenchymal stem cells (MSC) secrete neuroprotective molecules that may be useful as an alternative to cell transplantation itself. Our purpose was to develop different pharmaceutical compositions based on conditioned medium (CM) of adipose MSC (aMSC) stimulated by and/or combined with nicotinamide (NIC), vasoactive intestinal peptide (VIP), or both factors; and to evaluate *in vitro* their proliferative and neuroprotective potential. Nine pharmaceutical compositions were developed from 3 experimental approaches: (1) unstimulated aMSC-CM collected and combined with NIC, VIP, or both factors (NIC+VIP), referred to as the aMSC-CM combined composition; (2) aMSC-CM collected just after stimulation with the mentioned factors and containing them, referred to as the aMSC-CM stimulated-combined composition; and (3) aMSC-CM previously stimulated with the factors, referred to as the aMSC stimulated composition. The potential of the pharmaceutical compositions to increase cell proliferation under oxidative stress and neuroprotection were evaluated *in vitro* by using a subacute oxidative stress model of retinal pigment epithelium cells (line ARPE-19) and spontaneous degenerative neuroretina model. Results showed that oxidatively stressed ARPE-19 cells exposed to aMSC-CM stimulated and stimulated-combined with NIC or NIC+VIP tended to have better recovery from the oxidative stress status. Neuroretinal explants cultured with aMSC-CM stimulated-combined with NIC+VIP had better preservation of the neuroretinal morphology, mainly photoreceptors, and a lower degree of glial cell activation. In conclusion, aMSC-CM stimulated-combined with NIC+VIP contributed to improving the proliferative and neuroprotective properties of the aMSC secretome. Further studies are necessary to evaluate higher concentrations of the drugs and to characterize specifically the aMSC-secreted factors related to neuroprotection. However, this study supports the possibility of improving the potential of new effective pharmaceutical compositions based on the secretome of MSC plus exogenous factors or drugs without the need to inject cells into the eye, which can be very useful in retinal pathologies.

## 1. Introduction

Globally, retinal neurodegenerative diseases are a leading cause of blindness [1, 2]. Although the etiology and pathogenesis of most of these diseases are very different, many of them show common features due to the similarity of the retinal cellular response to different injuries. Thus, several therapeutic approaches have been proposed, including cell-based therapies dependent on neuroprotective mechanisms that could be adequate for many retinal neurodegenerative diseases [3]. Current research in stem cell therapy for retinal degenerative diseases is based on two main therapeutic approaches: (1) replacement of adult damaged cells by differentiating stem cells and (2) neuroprotection by using the paracrine stem cell properties [4–7]. For the latter purpose, mesenchymal stem cells (MSC) are the most frequently used stem cells [4, 6, 8], because they can provide trophic support for retinal cells via secretion of cytokines, growth factors, neurotrophic factors, proteins with angiogenic effects, inhibition of apoptosis, and modulation of the immune system and neuroinflammation [7, 9]. There are several sources of MSC, including bone marrow and adipose tissue. Bone marrow aspiration provides fewer MSC than does liposuction used to harvest adipose-MSC (aMSC) [9]. While aMSC collection is rarely the main reason for performing liposuction, the suctioned adipose tissue contains large amounts of aMSC that are usually treated as waste material and discarded, thus, disposing a potentially valuable resource [6, 10].

In a previous study made by our group, aMSC demonstrated the potential to partially rescue the human retinal pigment epithelium (RPE) cell line ARPE-19 from cell death induced by mitomycin C, an alkylating agent [11]. This result was enhanced by adding two drugs that play a significant role in cellular protection: nicotinamide (NIC), an amide active form of Vitamin B3 [12], and vasoactive intestinal peptide (VIP), a neuropeptide [13]. In the presence of NIC and VIP, aMSC stimulated the proliferation of mitomycin C damaged RPE cells and preserved neuroretinal (NR) explants from degeneration *in vitro* [14]. Those promising results were patented for neuroprotective effects of both drugs with the paracrine products secreted by aMSC (Patent WO/2015/079093). However, those outcomes were generated in cocultures, i.e., aMSC was always present with the target cells. Thus, this approach still presents several issues to be solved regarding biosafety and cell integration [7, 15].

On the other hand, a cell-free strategy based on a stem cell-conditioned medium (CM) constitutes a safer administration option while avoiding the potential risks associated with cell injection. Moreover, this approach presents noteworthy handling and storage advantages over living cells [16]. Thus, we hypothesize that similar protective effects can be obtained without the physical presence of the MSC themselves. However, it seems necessary to establish first which of the following circumstances determine the neuroprotective properties. That is, if this potential effect is (1) the consequence of the combination of neuroprotective potentials of the drugs and the aMSC secretome, (2) the increase of neuroprotective factors released by aMSC stimulated by these drugs, or (3) the combination of both mechanisms. Therefore, the purpose of this study was to develop and evaluate different pharmaceutical compositions (PhC) based on CM from aMSC stimulated by and/or combined with NIC, VIP, or both; and to analyze their effects in *in vitro* retinal degeneration models for assessing their potential as new therapeutic options for retinal degenerative diseases.

## 2. Materials and Methods

### 2.1. Cell Cultures

Human aMSC (StemPro® Human Adipose-Derived Stem Cells; Invitrogen; Paisley, UK) were seeded at a density of 5,000 cells/cm^2^ in 6-well culture plates (Corning Inc., Corning, NY, USA) and grown in low glucose Dulbecco's Modified Eagle Medium (DMEM) with GlutaMAX™ supplement, completed with 10% fetal bovine serum (FBS) and 2% antibiotics (100 U/ml penicillin and 100 *μ*g/ml streptomycin) (Gibco®, Invitrogen; Paisley, UK). The aMSC were grown under standard culture conditions of 5% CO_2_ at 37°C in a humidified cell culture incubator for 5 days, renewing the culture medium every 48 hours. At 80% confluence, aMSC was maintained in a serum-free medium for 24 hours to synchronize the cell cycle [17].

ARPE-19 cells were purchased from the American Type Culture Collection® (Manassas, VA, USA). This cell line (ATCC® number: CRL-2302™; Lot number: 59270158) was authenticated by the supplier (ATCC®) using the cytochrome C oxidase I gene (COI) assay (interspecies) and short tandem repeat (STR) profiling to verify the human unique DNA profile (intraspecies). ARPE-19 were grown in a mixture of DMEM and F-12 Nutrient Mixture (1 : 1) with GlutaMAX™ supplement, completed with 10% FBS and 1% antibiotics-antimycotics (100 U/ml penicillin, 100 *μ*g/ml streptomycin, 0.25 *μ*g/ml amphotericin B) (all Gibco®). For this study, cells were seeded at a cell density of 30,000 cells/cm^2^ in 96-wells culture plates (Corning Inc.) for 3 days until confluence and then maintained in serum-free medium for 24 hours to synchronize cell cycle.

### 2.2. Pharmaceutical Compositions (PhC)

NIC and VIP were purchased from Sigma-Aldrich® (St. Louis, MO, USA) and Calbiochem EDM Chemicals, Inc. (San Diego, CA, USA), respectively. Stock solutions were made, according to the manufacturer's instructions, at 10 mM and 5 *μ*M, respectively, and as used in our previous studies [14] (Patent WO/2015/079093).

**Figure 1:**
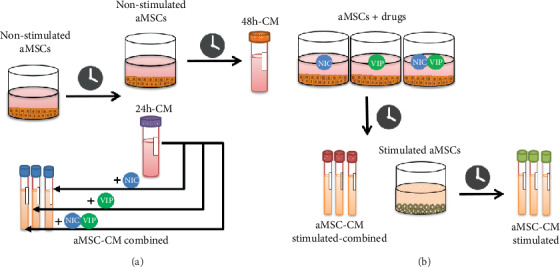
Development of pharmaceutical compositions (PhC) based on aMSC secretome and NIC and/or VIP. (a) aMSC were cultured under standard conditions for 24 hours. The CM were then collected, processed, and combined with selected drugs (NIC, VIP, or NIC+VIP) to be used as aMSC-CM combined (PhC 1). Nonstimulated aMSC were cultured for an additional 24 hours in fresh standard medium, and 48 h-CM was collected. (b) In parallel, aMSC were cultured under standard conditions with NIC, VIP, or NIC+VIP for 24 hours. The CM was collected and used as aMSC-CM stimulated-combined (PhC 2). Afterward, stimulated aMSC cells were cultured for an additional 24 hours in fresh standard medium without the addition of any other drugs, and the supernatants were collected and used as aMSC-CM stimulated (PhC 3). aMSC: adipose-derived mesenchymal stem cells; CM: conditioned medium, NIC: nicotinamide; VIP: vasoactive intestinal peptide.

After cell cycle synchronization, the aMSC were washed with phosphate-buffered saline (PBS; Gibco®) and exposed to 3 experimental conditions (Figure 1) to finally obtain 9 different PhC (Table 1):

1. aMSC were cultured in complete DMEM/F12 with GlutaMAX™ supplemented with 10% FBS and 2% antibiotics for 24 hours (Figure 1(a), left). Afterward, the supernatant was collected, centrifuged for 5 min at 1,000 rpm, and passed through a 0.2 *μ*m sterile syringe filter (Fisherbrand™, Loughborough, UK) to be used as CM (24 h-CM) or CM combined with NIC, VIP, or NIC+VIP just before use (PhC 1).
2. In parallel, aMSC were stimulated with NIC, VIP, or NIC+VIP prepared by dilution in complete DMEM/F12 with GlutaMAX™ supplemented culture medium (Figure 1(b), left). Subsequently, the supernatants of the stimulated aMSC containing the drugs were collected (PhC 2) and processed as described above
3. Finally, both stimulated and nonstimulated aMSC were washed with PBS and cultured in complete DMEM/F12 with GlutaMAX™ supplement culture medium for an additional 24 hours. The supernatants were collected, centrifuged, and filtered to be in use as 48 h-CM (Figure 1(a), right) and PhC 3 (Figure 1(b), right).

Fresh CM and PhC were prepared for each experimental procedure and stored at 4°C until used. Complete DMEM/F12 with GlutaMAX™ supplement culture medium was used as the control. Protocols used in this study to obtain CM and PhC were previously set up and optimized in our laboratory (preliminary studies, data not shown).

### 2.3. *In Vitro* ARPE-19 Cell Oxidative Stress Model

All PhC and CM were tested in an *in vitro* model of ARPE-19 cell oxidative stress. This model was set up by using glucose oxidase (GOx) from *Aspergillus niger* (Sigma-Aldrich®) to induce oxidative damage to the cells [18]. After cell cycle synchronization, ARPE-19 cells were exposed to different GOx concentrations (7-12 mU/ml in DMEM high glucose culture medium completed with 10% FBS and 1% antibiotics-antimycotic) for 24 hours. Untreated and treated cells with 0.001% benzalkonium chloride were used as negative and positive controls of cell death. ARPE-19 cell damage was assessed by using 3-(4,5-dimethylthiazol-2-yl)-2,5-diphenyltetrazolium bromide (MTT) cytotoxicity assay (Sigma-Aldrich®). Results were expressed as the percentage of living cells in each experimental condition to negative control (100% cell viability).

### 2.4. ARPE-19 Cell Proliferation Assays

After oxidative damage induction, ARPE-19 cells were washed with PBS and exposed to CM or PhC for 6 days. Cell proliferation assays were performed at days 0, 1, 3, and 6 using alamarBlue® (AbD Serotec, Oxford, UK). Complete DMEM/F12 with GlutaMAX™ supplement prepared with 10% alamarBlue® was added to each well and incubated at 37°C for 5 hours. Then, fluorescence was measured at 560 nm excitation and 590 nm emission wavelengths (SPECTRAmax M5, Molecular Devices, Sunnyvale, CA, USA). Cells were washed with PBS and then fresh CM or PhC was prepared with complete culture medium (in a proportion of 1 : 1). Three experiments in triplicate were performed in every experimental condition. Data collected immediately after oxidative damage were considered as “time 0” and used to normalize the obtained values.

### 2.5. Neuroretinal Explants Preparation and Organotypic Culture

Three fresh porcine eyes from animals aged 6-8 months old were obtained from the local slaughterhouse. NR explants were prepared following the protocol previously described by our group [19]. In summary, eyeballs were sectioned coronally just below the ora serrata, and the anterior segment and the vitreous were removed. The NR was carefully detached from the posterior segment, and the porcine cone-enriched visual streak (*area centralis*) was identified. Eight adjacent explants (5 × 5 mm) from this central area were obtained from each eye, avoiding those that contained visible blood vessels. The NR explants were laid over Transwell® membranes (12 mm diameter with 0.4 *μ*m pore Polycarbonate Membrane Insert; Corning Inc., Kennebunk, ME, USA) with the photoreceptors layer facing the membrane.

Just after NR explants preparation, they were exposed for 3 days to the CM or the PhC, (previously selected by the results of the ARPE-19 cell proliferation assays) prepared in 1 : 1 proportion, as done previously. The CM or the PhC level (0.5 ml) was maintained in contact with the support membrane beneath the explants (following the manufacturer's instructions) and renewed daily. One NR explant from each eye was directly processed to be used as the “time 0” control before culturing.

### 2.6. Neuroretinal Tissue Processing and Histological Characterization

After culture, NR explants were fixed for 2 hours in 4% paraformaldehyde (Santa Cruz Biotechnology, Inc., Dallas, TX, USA) and processed in an automatic tissue processor (Leica ASP300; Leica Microsystems, Wetzlar, Germany). One paraffin block per NR was made and 5 *μ*m thick sections were obtained with a microtome (Microm HM340E; Microm International GmbH part of Thermo Fisher Scientific, Walldorf, Germany).

For NR histological characterization, sections were dewaxed in xylene, rehydrated in a series of descending alcohols, rinsed in deionized distilled water, and stained with hematoxylin and eosin (H-E) (Sigma-Aldrich®). Stained sections were dehydrated in a series of ascending alcohols, cleared in xylene, mounted, and cover-slipped. NR samples were analyzed with a light microscope (DM4000B; Leica Microsystems), and images were acquired with a digital camera (DFC490; Leica Microsystems). Brightness and contrast were minimally adjusted, and the final figures were composed with Pixelmator 3.8 Phoenix (Pixelmator Team, Vilnius, Lithuania).

### 2.7. Neuroretinal Morphometry and Cell Counts

The NR parenchyma in H-E–stained sections was analyzed by measuring the total distance between the outer and inner limiting membranes (OLM and ILM) and the thickness of the outer and inner nuclear layers (ONL and INL). The number of cell nuclei per 100 *μ*m and the number of rows of nuclei in both nuclear layers was manually counted from the captured images. In all cases, the measurements were performed with ImageJ software (version 1.51 s; National Institute of Health, Bethesda, MD, USA) on 40X images from nonsequential NR sections (*n* = 6 per analysis).

### 2.8. Glial Cell Immunocytochemistry

NR sections for immunocytochemistry were dewaxed in xylene, rehydrated, and rinsed as described above. Afterward, they were washed with PBS and incubated in 0.01% trypsin (Sigma-Aldrich®) in PBS for 15 minutes at 37°C for antigen retrieval. Sections were rinsed and blocked in PBS with 4% goat serum and 0.3% Triton X100 (both from Sigma-Aldrich®) for 1 hour. Then, a combination of primary antibodies for vimentin (Vim, 1 : 25; Santa Cruz Biotechnology) and glial fibrillary acidic protein (GFAP, 1 : 500; Dako Denmark, Glostrup, Denmark) were applied. After incubating for 1 hour at room temperature (RT), sections were washed in PBS, and the corresponding species-specific secondary antibodies conjugated to Alexa Fluor 488 and 568 (green and red; 1 : 200; Life Technologies™) were added and incubated for 1 hour at RT in the dark. Thereafter, nuclei were counterstained with 4′,6-diamino-2-phenylindole dihydrochloride (DAPI, 10 *μ*g/ml; Molecular Probes, Eugene, OR, USA) for 5 minutes at RT and washed in PBS. Finally, samples were mounted in Dako Fluorescence Mounting Medium (Dako North America, Carpinteria, CA, USA) and cover-slipped. Control samples in which the primary antibodies were omitted and were processed in parallel, and no immunoreactivity was found in any case. Samples were analyzed with a light microscope equipped for epifluorescence (DM4000B, Leica Microsystems), and images were acquired with a digital camera (DFC490, Leica Microsystems). Brightness and contrast were minimally adjusted, and the final figures were composed with Pixelmator 3.8 Phoenix.

### 2.9. Statistical Analysis

All data were collected in a database created in Excel (Microsoft Office Excel 2016; Microsoft Corporation, Redmond, WA, USA) and subsequently analyzed by SPSS software (IBM SPSS statistic v23, SPSS Inc. Chicago, IL, USA). Values were expressed as mean ± standard error of the mean (SEM), and differences were considered statistically significant at *p* < 0.05 in all cases. Before statistical analysis, fluorescence data were normalized by log-transforming using the logarithm base two to obtain a normal distribution. Analysis of ARPE-19 cell proliferation at follow-up times was performed by repeated-measures analysis of variance (ANOVA) with Bonferroni corrections for multiple testing and Tukey's post hoc tests since the homogeneity of variance was validated (Levene's test). A comparison of means in other experiments with a normal distribution, where the homogeneity of variance was not validated (Levene's test), was performed by Welch test with Games-Howell post hoc test. Finally, in the case of nonparametric variables, group means were compared using the Kruskal-Wallis test. Graphs were made using Excel.

## 3. Results

### 3.1. Rescue of Oxidative Stressed ARPE-19 Cells by Pharmaceutical Compositions

**Figure 2:**
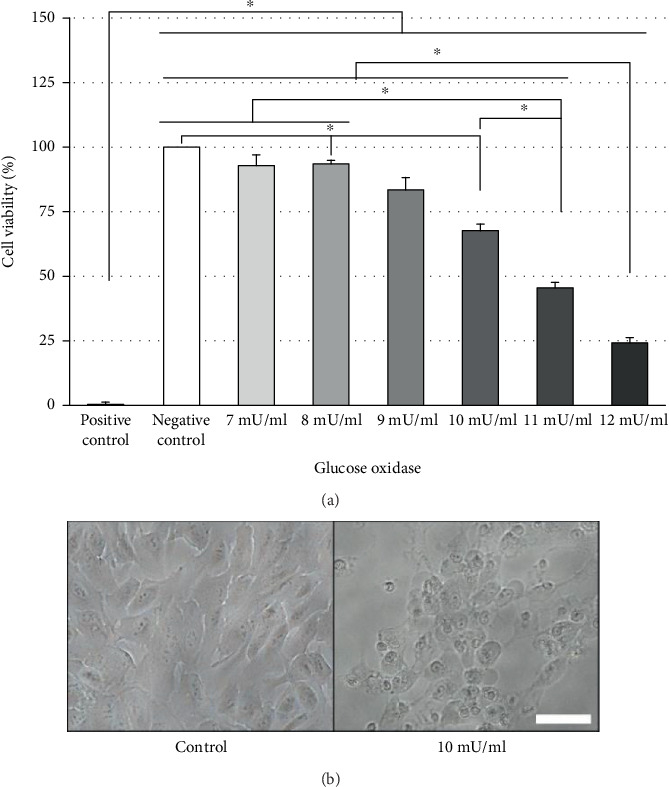
*In vitro* oxidative stress model. (a) MTT assay was used to evaluate cell viability. The H_2_O_2_ generated by glucose oxidase in the cell culture medium induced dose-dependent cytotoxicity in ARPE-19 cells after 24 hours of exposition. (b) Representative optical phase-contrast images of stressed ARPE-19 cells. Twenty-four hours of exposure to glucose oxidase induced changes in ARPE-19 cell shape, size, and nuclei. Bars represent the mean percentage ± SEM of cell viability (a). Statistical analysis: Welch test with Games-Howell post hoc test. ^∗^*p* < 0.05. (b) Scale bar = 50 *μ*m. Three experiments (*n* = 3) in triplicate were performed.

Initially, it was set up an *in vitro* ARPE-19 cell oxidative stress model to evaluate the rescue effects of the PhC. After GOx exposure, ARPE-19 cell viability, evaluated by MTT assay, decreased in a dose-dependent manner (Figure 2(a)). At higher concentrations (10-12 mU/ml), cell viability was significantly (*p* < 0.05) reduced to 68.2%-24.7%. Oxidative stress not only produced mortality, but it also induced changes in cellular morphology, such as cell rounding and shrinking (Figure 2(b)). Based on these data, 10 mU/ml GOx, which induced 31.8% mortality, was selected as the appropriate concentration to reliably produce damage in the ARPE-19 cells.

**Figure 3:**
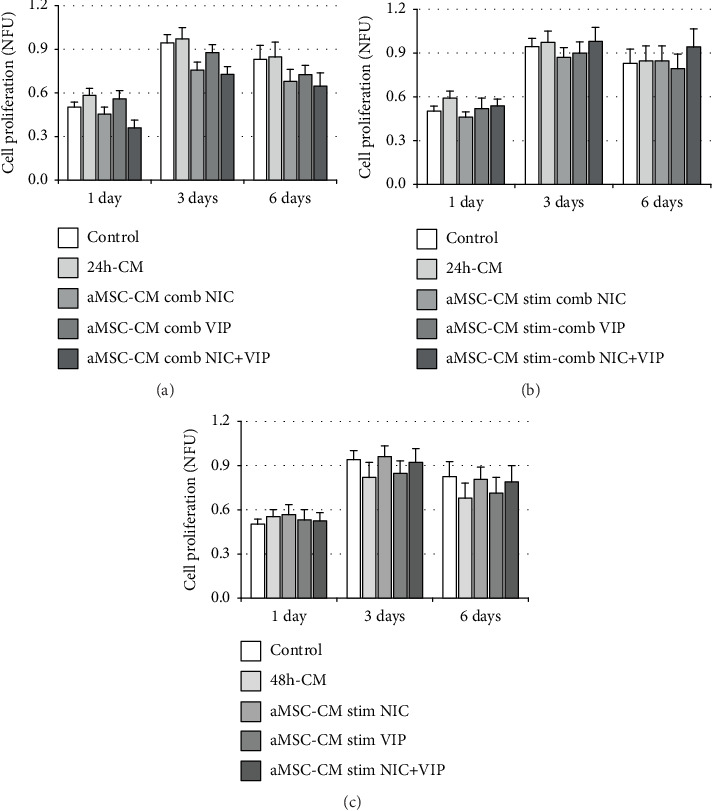
Cell proliferation in glucose oxidase stressed ARPE-19 cells cultured with the pharmaceutical compositions (PhC). Damaged ARPE-19 cell proliferation was measured using alamarBlue® at days 1, 3, and 6 of cell culture with the PhC. (a) aMSC-CM combined (PhC 1) with NIC, VIP, or NIC+VIP tended to reduce cell proliferation of glucose oxidase stressed ARPE-19 cells. While the effect was greatest in the presence of NIC+VIP, none of the differences were significant (*p* > 0.05). (b) aMSC-CM stimulated-combined (PhC 2) and (c) aMSC-CM stimulated (PhC 3) conditions tended to have higher better proliferation rates in glucose oxidase stressed ARPE-19 cells, though again, the differences were not significant. Bars represent the mean of normalized fluorescence unit (NFU) ± SEM. Statistical analysis: repeated measures ANOVA with Tukey's post hoc tests and Bonferroni corrections for multiple testing. Three experiments (*n* = 3) in triplicate were performed. aMSC: adipose-derived mesenchymal stem cells; CM: conditioned medium; NIC: nicotinamide; VIP: vasoactive intestinal peptide.

Stressed ARPE-19 cells cultured with 24 h-CM tended to increase cell proliferation (measured by alamarBlue®) in comparison with the control group (Figures 3(a) and 3(b)). However, when oxidative stressed ARPE-19 cells were exposed to PhC 1 with NIC, VIP, or NIC+VIP, cell proliferation tended to be lower than control and 24 h-CM groups (Figure 3(a)). This antiproliferative effect was more intense at culture day 1 when both drugs were added simultaneously. On the other hand, when damaged cells were exposed to the PhC 2 with NIC+VIP and PhC 3 with NIC (Figures 3(b) and 3(c)), they tended to have a better recovery from the oxidative injury, especially on days 3 and 6. Finally, stressed ARPE-19 cells cultured in 48 h-CM tended to have lower proliferation than did the controls or cells incubated with any of the PhC 3 (Figure 3(c)). While the tendencies for increased or decreased proliferation were consistently observed, the differences were not statistically significant.

### 3.2. Neuroretinal Morphology, Morphometry, and Cell Counts

Based on the results of the previous cell proliferation assays, CM, PhC 2, and 3 with NIC and NIC+VIP were chosen to be evaluated in NR organotypic cultures.

**Figure 4:**
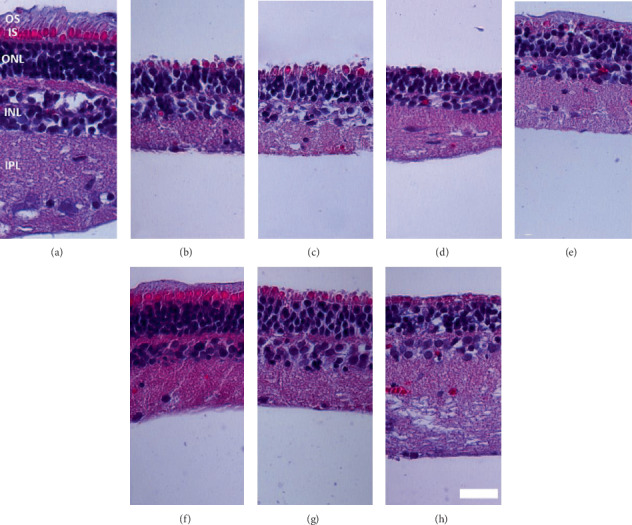
Photoreceptors modifications in porcine NR cultured for 3 days with aMSC-CM under stimulated and stimulated-combined conditions. Representative H-E–stained retina images from (a) freshly isolated NR, (b) control, (c) 24 h-CM, (d) 48 h-CM, (e) aMSC-CM stimulated-combined (PhC 2) with NIC and (f) with NIC+VIP, (g) aMSC-CM stimulated (PhC 3) with NIC and (h) with NIC+VIP. The photoreceptor outer segmentes were absent in all 3-day cultured samples, showing fibrotic-like membranes overlying the photoreceptor nuclei except those exposed to aMSC-CM stimulated-combined NIC+VIP (f), where photoreceptor outer and inner segments and ONL appeared better preserved. (g) NRs exposed to aMSC-CM stimulated NIC had also show photoreceptor outer and inner segments fragments and organized ONL. Scale bar denotes 25 *μ*m (*n* = 3 per group). aMSC: adipose-derived mesenchymal stem cells; CM: conditioned medium; H-E: hematoxylin and eosin; INL: inner nuclear layer; IPL: inner plexiform layer; IS: inner segments; NIC: nicotinamide; NR: neuroretina; ONL: outer nuclear layer, OS: outer segments; PhC: pharmaceutical compositions; VIP: vasoactive intestinal peptide.

To assess the structural preservation of porcine NR explants exposed to PhC, during 3 days, samples stained with H-E were evaluated (Figures 4(a)–4(h)). Freshly isolated NRs had a well-preserved, clearly defined, layered retinal structure and cellular morphology (Figure 4(a)). The photoreceptor outer and inner segments were only clearly apparent in fresh NR and those exposed to PhC 2 NIC+VIP (Figure 4(f)). These NRs had also better conservation of the layered structure than did the control and other experimental conditions (Figures 4(c)–4(h)). NRs cultured with PhC 3 NIC showed a fibrotic-like membrane overlying the photoreceptors (Figure 4(g)). NRs cultured in the PhC 3 had different effects according to which drugs were used. Samples exposed to PhC 3 with NIC showed a well-preserved ONL (Figure 4(g)), while those exposed to PhC 3 with NIC+VIP underwent some degeneration (Figure 4(h)).

After 3 days of culture, the effect of PhC on NR thickness in the H-E–stained sections was measured. The total thickness of freshly isolated NRs, 120.21 ± 5.02 *μ*m, was significantly higher than for the other culture conditions (*p* < 0.05 for PhC 3 NIC+VIP comparison; and *p* < 0.001 for the other comparisons). Each of the experimental cultures had a higher total thickness than the control group, 62.07 ± 1.09 *μ*m, but only for NRs exposed to PhC 3 with NIC+VIP, 96.61 ± 4.20 *μ*m, and 24 h-CM, 70.92 ± 1.99 *μ*m, the values were significantly thicker (*p* < 0.01 and *p* < 0.05, respectively). NRs exposed to PhC 3 with NIC+VIP were significantly thicker than those cultured in CM or other PhC (*p* < 0.05 for 48-CM comparison; and *p* < 0.01 for the other comparisons).

**Figure 5:**
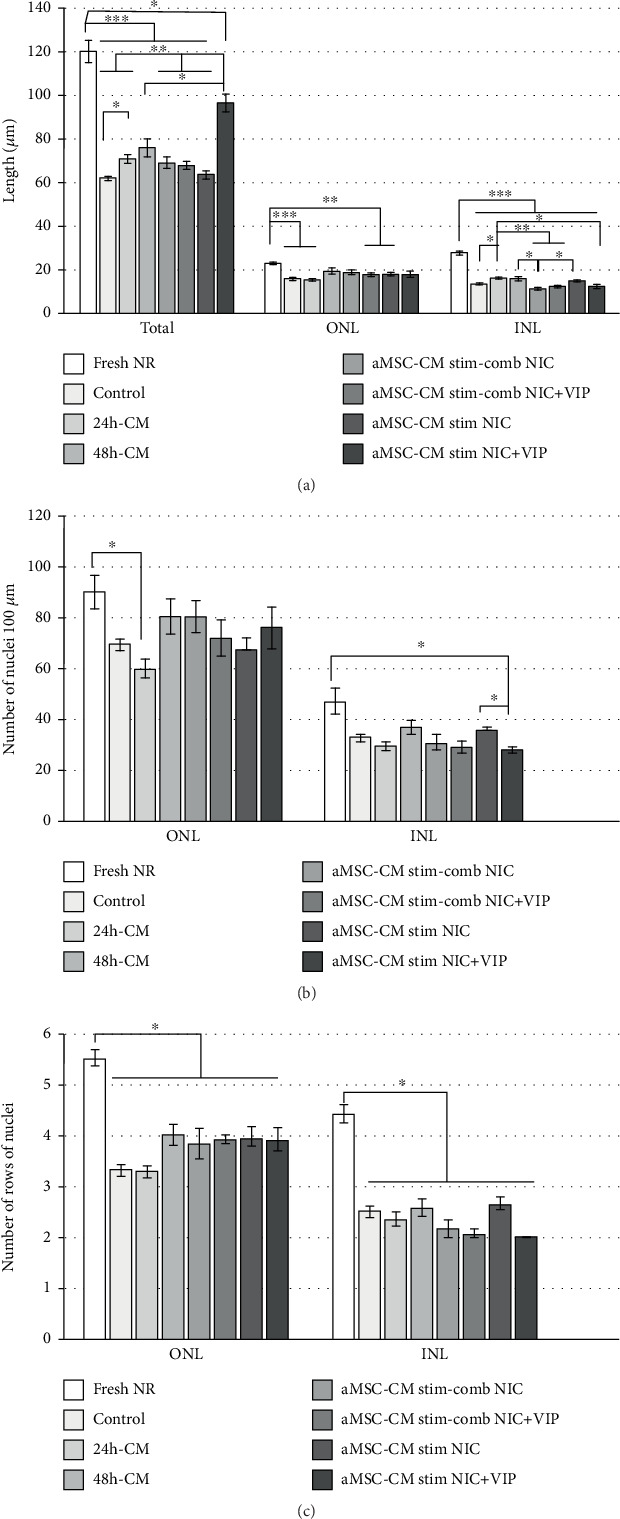
The neuroretinal thickness and cell counts. (a) Total and INL thicknesses were significantly decreased in cultured NRs in comparison with freshly isolated NRs. The ONL thickness was significantly reduced in cultures incubated with control, 24 h-CM, aMSC-CM stimulated combined (PhC 2) with NIC+VIP, and aMSC-CM stimulated (PhC 3) with NIC. (b) Similarly, the number of ONL and INL nuclei was lower in cultured samples than in fresh ones. However, the reduction was significant only in the ONL of NRs exposed to 24-CM and in the INLs exposed to aMSC-CM stimulated (PhC 3) with NIC+VIP. (c) The number of a row of nuclei in both ONL and INL was significantly reduced relative to fresh NR. Bars represent mean ± SEM. Statistical analysis: (a and b) Welch test with Games-Howell post hoc test (*n* = 3 per group); (c) Kruskal-Wallis test. ^∗^*p* < 0.05, ^∗∗^*p* < 0.01, ^∗∗∗^*p* < 0.001. aMSC: adipose-derived mesenchymal stem cells; CM: conditioned medium; INL: inner nuclear layer; NIC: nicotinamide; NR: Neuroretina; ONL: outer nuclear layer; PhC: pharmaceutical compositions; VIP: vasoactive intestinal peptide.

Detailed analysis of the nuclear layers showed that after 3 days of culture, the thickness of the ONL decreased significantly in the control culture, 24 h-CM, PhC 3 with NIC, and PhC 2 with NIC+VIP, in comparison with fresh NRs (*p* < 0.01 each; Figure 5(a)). However, only in the NRs cultured with the 24 h-CM medium, the number of nuclei was significantly reduced compared to freshly isolated NRs (*p* < 0.05; Figure 5(b)). Also, the count of rows of nuclei in this layer showed that it was significantly reduced relative to fresh NR (*p* < 0.05; Figure 5(c)). In all culture conditions, the INL was significantly thinner than for fresh NRs (*p* < 0.001 for all comparisons; Figure 5(a)). Moreover, the number of rows of nuclei in INL was also significantly lower in all samples cultured (*p* < 0.05; Figure 5(c)). In contrast to the ONL, the number of INL nuclei was significantly reduced only in the PhC 3 with NIC+VIP (*p* < 0.05; Figure 5(b)).

### 3.3. Glial Cells

**Figure 6:**
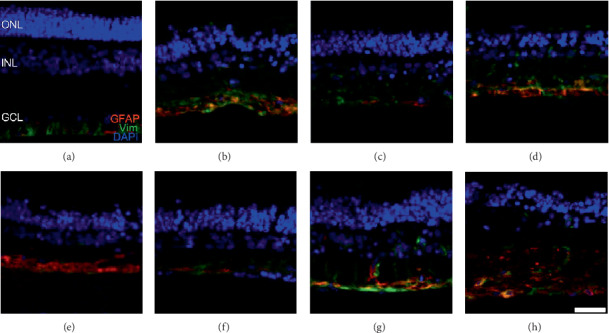
Glial cell activation. GFAP (red) and Vim (green) immunofluorescence in representative retina samples from (a) fresh NR, (b) control, (c) 24 h-CM, (d) 48 h-CM, (e) aMSC-CM stimulated-combined (PhC 2) with NIC and (f) with NIC+VIP, (g) aMSC-CM stimulated (PhC 3) with NIC and (h) with NIC+VIP (*n* = 3 per group). All explants were cultured for 3 days and had higher glial cell activation than fresh NR samples. However, this activation was lower in NR exposed to aMSC-CM stimulated-combined with NIC+VIP (f). Scale bar denotes 25 *μ*m. aMSC: adipose-derived mesenchymal stem cells; CM: conditioned medium; GCL: ganglion cell layer; GFAP: glial fibrillary acidic protein; INL: inner nuclear layer; NIC: nicotinamide; ONL: outer nuclear layer; PhC: pharmaceutical compositions; Vim: Vimentin; VIP: vasoactive intestinal peptide.

Gliosis is an indicator of glial cell activation, a process that occurs in response to neuroretinal damage, which can be evaluated by Vim and GFAP immunoreactivity in neuroretina cultures [20, 21], associated with modifications in the intermediate filaments of astrocytes and Müller cells [22]. We assessed gliosis in fresh NRs (Figure 6(a)) and those cultured in control medium, CM, and PhC (Figures 6(b)–6(h)). In fresh NRs and those exposed to PhC 2 with NIC+VIP, both Vim and GFAP were located in and limited to the neuroretinal innermost layers (Figures 6(a) and 6(f)). The immunoreactivity was increased in the cultures incubated with 48 h-CM (Figure 6(d)) and each of the groups of the PhC 3 (Figures 6(g) and 6(h)) where proteins were detected from the ILM to the INL. In the other culture conditions, the immunoreactivity was only detected from the ILM to the inner plexiform layer (Figures 6(b), 6(c), and 6(e)).

## 4. Discussion

Currently, there is an increasing interest in stem cell-based therapies as potential treatments for retinal degenerative diseases. These cell therapies' usefulness relies on the replacement of the damaged cells and/or neuroprotective effects through MSC secretome [4–7]. However, there are still several difficulties that must be addressed regarding the safety of stem cell injections in patients before clinical use. Potential risks associated with cell-based applications include engraftment at an ectopic location, inappropriate differentiation, aggregate formation, and others [15]. The use of MSC-CM may make possible a safe alternative to stem cell transplants, and therefore, an option to use their therapeutic properties while avoiding the associated potential risks. Besides, handling and storage of MSC secretome are remarkably advantageous as compared to living cells, for the clinical practice [16]. Considering all the abovementioned benefits of the use of cell-free strategies and the previously demonstrated neuroprotective effect of aMSC-CM [23–26], we aim at developing effective procedures that take advantage of the stem cell secretome without requiring the injection of living stem cells into the patient's eye.

This study showed that PhC, specifically PhC 2 with NIC+VIP, enhances the *in vitro* survival of damaged ARPE-19 cells and slow down retinal degeneration. Human cell line ARPE-19 retains many of the functional and structural properties characteristic of RPE *in vivo* [27, 28]; and organotypic NR cultures are adequate tools to evaluate neurons and glial cells modifications, close to the *in vivo* retina [29–32]. Thus, although these are *in vitro* models, they allow the screening of PhC under controlled laboratory conditions, and therefore reducing the number of living animals for experimental purposes.

We have previously shown that aMSC can partially rescue damaged RPE [11], and that cell survival is improved with the use of the neuroprotective drugs VIP and NIC, at 5 *μ*M and 10 mM, respectively [14] (Patent WO/2015/079093). The VIP concentration used in those studies had been previously used to induce the differentiation of aMSC toward RPE cells [33]. Although this concentration was higher than the usual VIP doses tested for neuroprotection [34], our previous studies using VIP with the presence of aMSC support the use of 5 *μ*M concentration in the study presented here. On the other hand, the NIC concentration (10 mM) has demonstrated neuroprotective properties both in the culture of cortical cells [35] and over ischemic rabbit retina [36]. As well as VIP, our previous studies confirmed the neuroprotective effect of NIC at the mentioned concentration with the presence of aMSC [14].

Those studies were performed by coculturing with the aMSC. Nevertheless, in the current study, 9 PhC based on aMSC secretome and NIC, VIP, or both, were developed. Although the neuroprotective effects of each of these drugs have been extensively described on RPE cells and neuroretina [34, 36–41], the current study focused on determining the most effective way of interaction between NIC and/or VIP and the aMSC, for cell protection. Thus, 3 different experimental approaches, stimulation, combination, or both, were used to develop the PhC. However, aMSC consumes nutrients of the culture medium during the conditioned period. Therefore, it was necessary to supplement the PhC to provide the required nutrients with fresh culture medium (1 : 1) to adequately maintain the cells during cultures. Nevertheless, this led to a reduction of the final concentration.

In previous studies, we had used a mitomycin C treated RPE cell culture to evaluate aMSC rescue potential [11]. Nonetheless, mitomycin C is an alkylating agent that inhibits the DNA synthesis [42], and therefore, an external agent that is not involved in the pathogenesis of retinal degeneration. Hence, in this study, an *in vitro* model of oxidative stress in RPE cells was developed and characterized to evaluate the potential to enhance cell survival of the different developed PhC. The eye, due to its local exposure to light and the high level of oxygen chromophores, is a particularly susceptible target to oxidative damage [43–47], which is one of the mechanisms involved in the pathogenesis of retinal degeneration [3, 43, 45, 48]. The RPE cells have crucial roles in the eye, and they need to be protected against, continuously *in vivo* generated, reactive oxygen species (ROS) and other potentially toxic agents. Therefore, H_2_O_2_, the most important ROS, was continuously generated in the culture medium by the enzyme GOx. This enzyme uses the glucose supplement present in the cell culture medium as a nutrient for cell survival. This ensured a sustained delivery of H_2_O_2_ in cell culture over a long period, closely resembling the physiological situation in comparison with the pulse delivery of exogenous H_2_O_2_ [18, 49] used in other studies [50–55]. This *in vitro* model had been previously used to research into the oxidative stress on the proteasome [56] and evaluate the protective potential of cellular melanosomes [49] and plant extracts [57]. Under these culture conditions, lower concentrations of GOx did not induce significant cell death, while higher concentrations exhibited dose-dependent cytotoxicity. These outcomes may be because the antioxidant defense of ARPE-19 cells is only effective against low concentrations of ROS [18, 57].

In this study, the rescue effect of PhC on stressed ARPE-19 cells was evaluated using alamarBlue®, a redox indicator which allows obtaining several cell proliferation measurements throughout the follow-up period without cell damage. This reactive agent is widely accepted for evaluating cellular health status and metabolic function [58]. Moreover, it has been demonstrated that the alamarBlue® fluorescence results are correlated with the cell numbers estimated by direct cell-counting [59].

The addition of NIC and VIP to aMSC-CM exhibited an antiproliferative effect on damaged ARPE-19 cells in this study. Our previous studies (Patent WO/2015/079093) had shown that NIC, VIP and, overall NIC+VIP presented an antiproliferative effect in damaged ARPE-19 cells, which supports the current results. VIP is also known to inhibit RPE proliferation [37], an effect that is intensified in damaged cells. On the other hand, NIC is an inhibitor of the enzyme polyADP-ribosepolymerase-1 (PARP-1) [12, 60] that plays an important role in the regulation of cell death and DNA repair [12] and the tolerance of RPE cells to oxidative injury [61]. Besides, NIC has been used to induce the differentiation of ARPE-19 cells [38]. This fact could be related to the antiproliferative effect observed in this study since some differentiated ARPE-19 genes involved in cell proliferation are downregulated [62]. Consequently, we discarded the hypothesis of a synergistic effect between unstimulated aMSC-secretome and either NIC or VIP.

When the oxidatively injured cells were exposed to the PhC 2 or 3, ARPE-19 proliferation tended to increase, indicating that cells were recovering from oxidative stress. Interestingly, the PhC 2, in which not only the secretome of stimulated aMSC was present, but also the drugs, showed improved proliferation, possibly due to the enhancement of antioxidative properties. These could be the consequence of an increase in the concentration of certain factors in the aMSC-secretome, in the secretion of new effective molecules or both. Moreover, during the stimulation period, some amount of NIC could be converted to nicotinamide adenine dinucleotide (NAD^+^) [12, 60], a molecule with the potential to modulate MSCs function, including cytokine release [63], and therefore, it could modify the aMSC secretome composition. However, further studies are necessary to clarify that point and evaluate higher doses of these PhC.

Based on the effects observed in ARPE-19 proliferation assays, we selected PhC 2 and 3 with NIC and with NIC+VIP to evaluate their potential neuroprotective effects on NR cultures. Organotypic NR is considered a very useful *ex vivo* model of retinal degeneration and active gliosis [20, 21] for retinal research [29] because they maintain the complex tissue structure and the connections between layers and cells [29–32]. These cultures have been used as models of retinal degeneration [20, 21, 64, 65] to evaluate novel therapeutic strategies, including aMSC [14, 66], or the cytotoxicity of ophthalmic medical devices [65]. Although human organotypic retinal cultures are the most appropriate tissue to resemble human ocular diseases [32, 67], their use poses significant disadvantages, such as the limited tissue availability and the donor-to-donor variability [32]. Because of these limitations, in this study, we used NR explants from porcine origin. Furthermore, the pig eye is very similar to the human being in size and also in retinal structure and ultrastructure [68, 69].

Previous studies have reported the neuroprotective effect of aMSC on different types of retinal cells [23, 24, 70–72]. In the current study, NR general morphology was better preserved in cultures with PhC 2 with NIC+VIP, including photoreceptor outer and inner segments and ONL. However, this well-preserved morphology was not correlated with the best-preserved neuroretinal thickness. The highest retinal thickness was obtained with PhC 3 with NIC+VIP, but relevant degenerative changes in the NR morphology and glial cell activation were observed. This degeneration may be related to an increase in the retinal thickness [20, 73, 74], which could explain the results observed. NR cultured with PhC 3 with NIC also showed suitable preservation of the NR layers, although in this case, outer segments were not identifiable. These outcomes could be due to VIP-induced neuroprotective factors released by the glial cells [13]. The presence of both VIP receptors [75] and VIP-producing neurons [76, 77] has been described in the retina. This supports the action of VIP on this ocular tissue. Besides, VIP neuroprotective effects have been previously observed in retina degeneration both *in vitro* [34] and *in vivo* [39, 40]. Action of NIC in several pathways is involved in neuronal injury protection [35, 78], and its protective effects have been observed in an *in vitro* rabbit retina model [36]. Therefore, in these two PhC, the drugs may improve the potential neuroprotective effect of the aMSC-CM on photoreceptors.

Regarding gliosis, aMSC stimulated-combined with NIC+VIP reduced the activation of glial cells in organotypic NR cultures. The potential of aMSC-CM to counteract the activation of glial cells is enhanced through cell stimulation with inflammatory cytokines [25, 26]. However, in the present study, the reduction of reactive gliosis was due to not only the aMSC secretome but also through the action of the addition of the drugs. VIP not only stimulated glial cells to release neuroprotective factors but also is involved in the physiology and metabolism of these cells [79]. On the other hand, it has been shown that there is a correlation between the activation of the enzyme PARP-1 and the glial cell activation [80]. Furthermore, inhibitors of this enzyme, such as NIC, reduce reactive gliosis in the degenerative retina [41]. Therefore, the factors secreted in aMSC stimulated-combined with both drugs seem to exhibit a synergistic effect not present in the other tested conditions.

However, to continue advancing in this potential treatment, it is necessary to discern if the results presented here are due to an increase in the concentrations of certain aMSC-secreted factors alone or to the secretion of new bioactive molecules by the aMSC in the presence of NIC and VIP. Therefore, an in-depth proteomic analysis to determine which of the PhC's components and mechanisms of action involved in the effects observed will be necessary. To complete these analyses, they should include a study of the stability of NIC and VIP under the *in vitro* conditions used to develop the PhC. This information will be especially relevant for the latter since there is little available information about its stability [81]. Knowledge of these elements will facilitate the approach of cell therapy using their secretome-based PhC, which not only represents a safer alternative to living cell transplant [16] but also allows more quality control and facilitates dosing [25].

## 5. Conclusions

In conclusion, our findings presented here suggest that it is possible to enhance the proliferative and neuroprotective properties of the aMSC secretome through stimulation by neuroprotective drugs, such as NIC and VIP and/or combination with them. To our knowledge, this is the first study that develops PhC based on aMSC secretome and these drugs. In the present study, we tested only one concentration of each PhC. However, the observed outcomes allow making a preliminary selection of the potential effective PhC. Higher doses may be necessary to improve significantly the rescue and neuroprotective effects obtained. This will be one of the targets for further studies. Furthermore, new studies are necessary to specifically identify and quantify the factors secreted by stimulated aMSC related to stressed RPE cell survival and neuroretina preservation *in vitro*. However, our approach points out the potential of new effective PhC based on the secretome of MSC stimulated by and combined with exogenous factors or drugs, without the need to inject cells into the eye, which can be very useful in retinal pathologies.

## Figures and Tables

**Table 1:** Pharmaceutical compositions.

Acronym	aMSC-CM	Drugs present
PhC 1 with NIC	Combined	NIC
PhC 1 with 1 VIP	Combined	VIP
PhC 1 with 1 NIC+VIP	Combined	NIC+VIP
PhC 2 with NIC	Stimulated-combined	NIC
PhC 2 with 2 VIP	Stimulated-combined	VIP
PhC 2 with NIC+VIP	Stimulated-combined	NIC+VIP
PhC 3 with NIC	Stimulated	None
PhC 3 with VIP	Stimulated	None
PhC 3 with NIC+VIP	Stimulated	None

aMSC: adipose-derived mesenchymal stem cells; CM: conditioned medium; NIC: nicotinamide; PhC: pharmaceutical compositions; VIP: vasoactive intestinal peptide.

## Data Availability

The data used to support the findings of this study are available from the corresponding author upon request.
